# A Limitation of Hartmann-Shack System in Measuring Wavefront Aberrations for Patients Received Laser Refractive Surgery

**DOI:** 10.1371/journal.pone.0117256

**Published:** 2015-02-18

**Authors:** Ying Wu, Ji C. He, Xing T. Zhou, Ren Y. Chu

**Affiliations:** 1 Eye & ENT Hospital of Fudan University, Shanghai, China; 2 New England College of Optometry, Boston, Massachusetts, United States of America; Sun Yat-sen University, CHINA

## Abstract

**Purpose:**

To explore the relationship between ablation parameters of myopic laser surgery and measurement area of wavefront aberration (WA) with Hartmann-Shack wavefront sensor.

**Methods:**

58 subjects undergone myopic laser surgeries and 74 uncorrected myopic subjects were enrolled in this experiment. The laser ablation parameters were obtained from surgical records, which included spherical error (Rx), depth, and optical zone (OZ) of ablation. The measured area of WA was tested by the WASCA, and the real pupil size was tested by Pentacam. The corneal eccentricity (E value) and curvature was also measured with the Pentacam. All the measurements were performed under mydriatic condition.

**Results:**

For uncorrected myopic eyes, the measured area of WA was similar with the real pupil size. But for the corrected eyes, the measured area of WA was smaller than the real pupil size with a mean difference of 0.66 ± 0.54 mm for moderate myopia (t = 6.45, p < 0.0001) and 1.76 ± 0.55 mm for high myopia (t = 18.92, p < 0.0001), but not for mild myopia. The Rx (t = -3.20, p = 0.0017), OZ (t = 64.4, p < 0.0001) and postoperative corneal E value (t = 2.52, p = 0.017) were the independent factors of measured area of WA. Measured area of WA = -0.81*Rx + 1.13*OZ + 0.49*postoperative corneal E value (r^2^ = 0.997).

**Conclusions:**

The WASCA has a limitation in measuring wavefront aberration over the whole pupil area when it’s used for patients received myopic laser surgery. The measured area is smaller than the real pupil size and depends linearly on ablation depth, optical zone and corneal eccentricity.

## Introduction

Wavefront aberrometry has been widely used in both optometry and ophthalmology areas since the modern version of wavefront sensor was introduced into eye research about 20 years ago[1,2]. The newly developed wavefront sensor provides capability to assess higher order aberrations of the eye, in addition to the lower order spherical error and astigmatism as measured with conventional refractometry[3,4]. Currently, several models of wavefront sensors have been employed in clinical practices for routinely diagnosing optical defects of the eye and also for evaluating optical effect in clinical treatments, such as laser refractive surgery[5,6], cataract surgery[7,8] and contact lenses[9,10].

So far, Hartmann-Shack wavefront sensor perhaps is the most widely used technique in clinical offices, due to its fast speed of measurement. A key part in the Hartmann-Shack sensor system is its microlenslet, which is consisting of a two dimensional array of micro lenses[3,4]. The lenslet is optically conjugated with the entrance pupil of the eye, and its function is to sample the whole area of the pupil for the light beam reflected back from a retinal point light source, which was first optically projected onto the retina. A numbers of small image spots of the retinal light source, depending on density of microlenses, are then formed by the lenslet, and they are received by a CCD camera located on the focal plane of the lenslet. The positions of the small image spots are used to analyze the wavefront aberrations of the eye, because deviations of the image spots from their ideal positions are directly proportional to wavefront error of the eye at the corresponding pupil area sampled by the microlenses. It is therefore always desired that the whole pupil area could be sampled so that the wavefront aberrations within the whole pupil area could be measured.

Excimer laser refractive surgery is an effective and precise method for correcting myopia, but it inevitably induces higher-order aberrations[11–15]. The induced higher-order aberrations impair visual quality especially under scotopic condition when the pupil size is relatively large[16–18]. In order to evaluate optical and visual effects of a laser refractive surgery on post-operative eyes, it is necessary to assess not only residual lower-order refractive error but also higher-order aberrations as well.

However, during our experience of measurement of higher-order aberrations for the eyes undergone high myopic excimer laser surgery with the Hartmann-Shack wavefront sensor (WASCA, Carl Zeiss Meditec, Oberkochen, Germany), it has been very often to notice that the measured area of wavefront aberration (WA) is much smaller than the measured area for the normal eye even if the pupil was fully dilated. This makes problems for the evaluation of optical effects caused by high myopic laser surgery, because it’s impossible to obtain the wavefront aberrations within the whole pupil area. The other problem encountered in practice is that the wavefront-guided enhancement could not be performed if the measured area of WA is too small[19].

As far, no systematical study on the measurement area problem of the Hartmann-Shack wavefront sensor has been reported in literature. Purpose of this study was to investigate the relationship between the measured are of WA with Hartmann-Shack wavefront sensor and ablation parameters of myopic laser refractive surgery for a group of myopic patients.

## Materials and Methods

This case series was performed at Eye & ENT hospital of Fudan University, Shanghai, China. The study followed the tenets of the Declaration of Helsinki and was undertaken with the understanding of each subject. The written consent was obtained from each subject to participate in this study. The study was approved by Eye & ENT Hospital’s ethic committees for the Protection of Human Subjects.

### Subjects

58 myopic subjects undergone uneventful laser in situ keratomeliusis (LASIK) to correct myopia and astigmatism 6 months ago and 74 uncorrected myopic subjects were enrolled in this study. The subject’s ages were from 18 to 38 years (mean ± S.D., 24.6 ± 5.0 years). For each subject, only one eye was randomly selected to be examined. The eyes were divided into mild, moderate, and high myopic groups according to their preoperative spherical errors. The mild myopia was lower than -3.0 diopters (Ds) preoperatively, the moderate myopia was from -3.0 Ds to -6.0Ds preoperatively, and the high myopia was higher than -6.0 Ds preoperatively. All the eyes were free of ocular diseases and ocular trauma.

The LASIK procedure included the creation of corneal flap with the VisuMax femtosecond laser (Carl Zeiss Meditec) and the stromal ablation with the MEL-80 Excimer Laser workshop (Carl Zeiss Meditec) using the tissue-saving mode. The parameters of laser ablation were obtained from surgical records, which included spherical error (Rx) of ablation, astigmatism of ablation, spherical equivalent (SE) of ablation, optical zone (OZ) of ablation and depth of ablation. For each eye, the postoperative visual acuity was no less than 20/25 and the residual refractive error was within ± 0.50 D.

### Procedures

**Fig 1:**
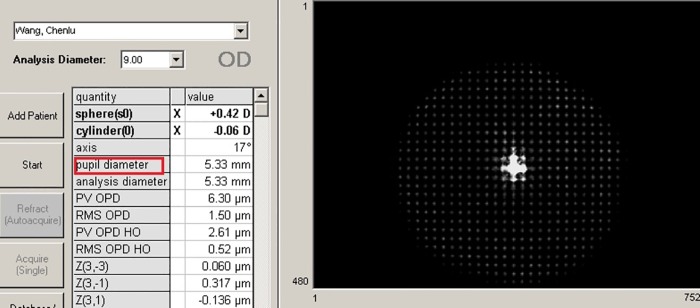
Measured area of wavefront aberration marked with a red rectangular frame.

All the measurements were performed under mydriatic condition. Specifically, each eye received the tropicamide phenylephrine eye drops (Santen Pharmaceutical (China) Co., Ltd., Jiangsu Province, China) three times with the interval of 5 minutes between each time. After the pupil had been fully dilated, the wavefront aberrations were measured with the Hartmann-Shack wavefront sensor (WASCA, Carl Zeiss Meditec, Oberkochen, Germany) using the conventional mode. The measured area of WA was automatically provided from the instrument (**Fig. 1**). Each measurement was repeated three times.

The conventional mode of WASCA automatically compensates the spherical error of the eye, therefore the relatively sharp image spots could be obtained for further analysis of wavefront aberrations. Because the myopic laser surgery inevitably induces a great difference in the amount of spherical error between the central ablated area and the peripheral area, it could make problems for the WASCA to compensate the spherical error for the whole pupil area. We tested the effect of spherical error compensation on the measured area of WA on two eyes. After the conventional measurement with the WASCA, the amount of spherical error compensation was manually changed by sliding the measuring bar with a step of -1.0 D from its conventional position to -8.0 D. The measured area of WA with different spherical error compensation was recorded using the “free running” mode.

The built-in CCD camera of Pentacam (OCULUS Optikgerate GmbH, Wetzlar, Germany) was used to measure the real pupil size. The Pentacam was also performed to measure corneal E value and Km. Each measurement was repeated three times.

### Statistical analysis

All the statistical analyses were performed using SAS software (version 8.2; SAS Institute, Cary, NC). The paired T test was used to compare the difference between the measured area of WA and the real pupil size for each group. The Pearson correlation analyses were performed between the measured area of WA and each parameter of laser ablation for all of the eyes. The ablation parameters included Rx, astigmatism, SE, OZ, and depth of ablation. Pearson correlation analyses was tested for the relationship between the measured area of WA and corneal E value and Km. A multiple stepwise regression analysis was used to find the independent factors to the measured area of WA and create a mathematical model out of them.

## Results

### Measured area of wavefront aberrations for uncorrected myopic eyes

The measured area of WA for uncorrected myopic eyes showed no significant difference from their real pupil size in each group (all p > 0.05, **Table 1**).

**Table 1:** Characteristics of uncorrected myopic eyes (mean ± S.D.).

characteristics	mild myopia	moderate myopia	high myopia
number	14	26	34
spherical error (D)	-2.25 ± 0.64	-4.77 ± 0.93	-8.60 ± 1.35
Astigmatism (D)	-1.02 ± 0.43	-1. 23 ± 0.34	-1.65 ± 1.51
spherical equivalent (D)	-2.69 ± 0.59	-5.38 ± 0.60	-9.22 ± 1.44
measured area of wavefront aberration (mm)	7.43 ± 0.44	7.44 ± 0.38	7.30 ± 0.33
real pupil size (mm)	7.52 ± 0.54	7.48 ± 0.35	7.39 ± 0.41

### Reduction of measured area of wavefront aberrations for corrected myopic eyes

**Fig 2:**
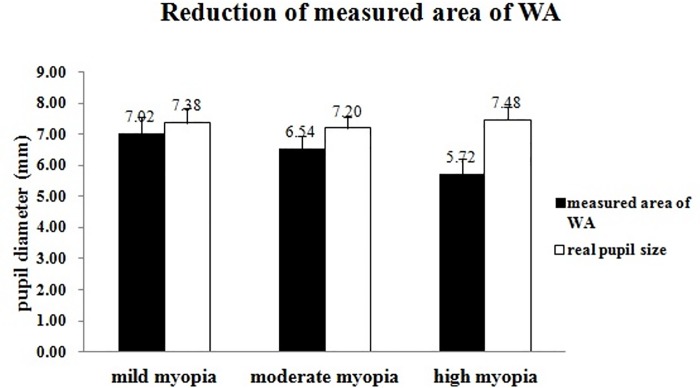
Reduction of measured area of wavefront aberration (WA) compared to real pupil size for different refractive groups. Error bars indicate one standard deviation from the mean. * indicates p < 0.05.

The preoperative and postoperative characteristics for corrected myopic eyes were listed in **Table 2**. Fig. 2 shows that the measured area of WA was significantly smaller than the real pupil size for moderate myopia (n = 19, t = 6.45, p < 0.0001), and high myopia (n = 29, t = 18.92, p < 0.0001), but not for mild myopia (n = 10, t = 1.56, p = 0.076). The mean reduction of measured area of WA as compared to real pupil size was 0.35 ± 0.22 mm for mild myopia, 0.66 ± 0.54 mm for moderate myopia, and 1.76 ± 0.55 mm for high myopia.

**Table 2:** Characteristics of corrected myopic eyes (mean ± S.D.).

characteristics	mild myopia	moderate myopia	high myopia
number	10	19	29
spherical error (Rx) of ablation (D)	-2.20 ±0.75	-4.25 ± 0.75	-8.05 ± 1.50
astigmatism of ablation (D)	-1.00 ± 1.02	-1.24 ± 1.21	-1.73 ± 1.55
spherical equivalent (SE) of ablation (D)	-2.63 ± 0.81	-4.71± 0.54	-8.85± 1.50
optical zone (OZ) of ablation (mm)	6.58 ± 0.12	6.56 ± 0.10	6.25 ± 0.31
depth of ablation (μm)	79.21 ± 15.86	94.40 ± 15.20	139.16 ± 17.40
postoperative corneal eccentricity	-0.45 ± 0.31	-0.60 ± 0.17	-0.96 ± 0.16
postoperative corneal curvature (D)	41.15 ± 1.22	39.50 ± 1.10	37.18 ± 1.68
measured area of wavefront aberration (mm)	7.02 ± 0.54	6.54 ± 0.41	5.72 ± 0.52
real pupil size (mm)	7.38 ± 0.48	7.20 ± 0.35	7.48 ± 0.52

### Correlation between the measured are of WA and the ablation parameters for the corrected myopic eyes

A significant linear relationship was found between the measured area of WA and several parameters of laser ablation, which included Rx, SE, depth, and OZ of ablation (**Table 3**). The measured area of WA was also significantly correlated to postoperative corneal E value (r = 0.55, p < 0.0001). A multiple stepwise regression analysis was performed and found 3 independent factors for the measured area of WA, which were Rx, OZ of ablation, and postoperative corneal E value **(Table 4**). The mathematical model was Measured area of WA = -0.81*Rx of ablation + 1.13*OZ of ablation + 0.49*postoperative corneal E value (r^2^ = 0.997).

**Table 3:** Pearson correlation analyses between the measured area of wavefront aberration and the parameters of laser ablation.

parameters of laser ablation	r value	p value
spherical error (Rx) of ablation	-0.78	<0.0001
spherical equivalent (SE) of ablation	-0.78	<0.0001
depth of ablation	-0.66	<0.0001
optical zone (OZ) of ablation	0.65	<0.0001
astigmatism of ablation	-0.12	0.24

**Table 4:** Multiple stepwise regression analysis for the measured area of wavefront aberration.

independent factors	t value	p value
spherical error (Rx) of ablation	-3.20	0.0017
optical zone (OZ) of ablation	64.4	<0.0001
postoperative corneal E value	2.52	0.0176

### The effect of spherical error compensation on the measured area of wavefront aberrations

**Fig 3:**
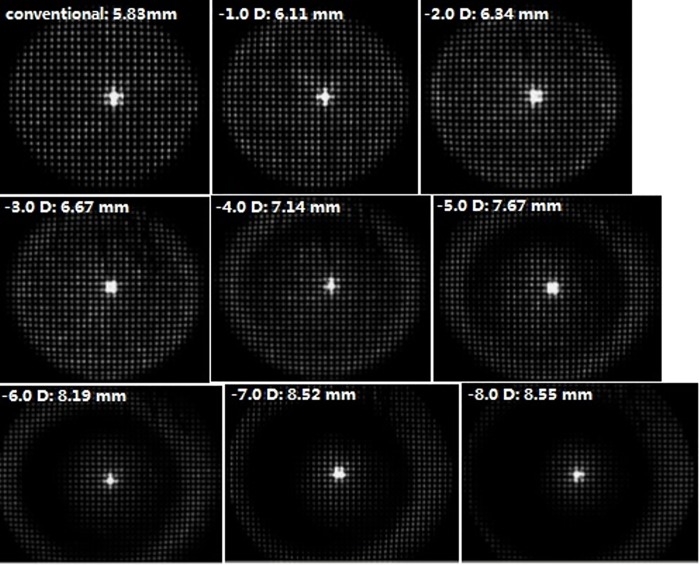
Influence of adjusting spherical error compensation on measured area of wavefront aberration and the impairment of image spots.

The spherical error compensation test showed a great influence of spherical error compensation on the measured area of WA for the postoperative patients. One patient received excimer laser ablation with the Rx of -8.75 D and the OZ of 5.75 mm. Under mydriatic condition, the real pupil size measured by the Pentacam was 9.00 mm. But, the measured area of WA was 5.83 mm by using conventional mode of WASCA **(Fig. 3**). With the adjustment of spherical error compensation, the measured area of WA systemically increased toward the real pupil size, but at the price of impairment of image spots (**Fig. 3**). As a result, the measurement of WA within 5.5 mm area became more and more inaccurate with the adjustment of spherical error compensation (**Table 5**).

**Table 5:** The changes of wavefront aberration with the adjustment of spherical error compensation.

spherical error compensation	measured area (mm)	defocus (D)	tRMS (μm)	hRMS (μm)	Z(3,-3) (μm)	Z(3,-1) (μm)	Z(3,1) (μm)	Z(3,3) (μm)	Z(4,-4) (μm)	Z(4,-2) (μm)	Z(4,0) (μm)	Z(4,2) (μm)	Z(4,4) (μm)
conventional	5.83	-0.36	2.35	0.49	0.06	0.32	0.30	-0.09	-0.04	0.00	-0.98	-0.06	-0.07
-1.00 D	6.11	-0.60	2.51	0.47	0.07	0.27	0.48	-0.10	0.00	0.01	-0.95	-0.05	-0.10
-2.00 D	6.34	-0.68	2.64	0.48	0.04	0.36	0.47	-0.10	-0.04	0.02	-0.96	-0.09	-0.13
-3.00 D	6.67	-0.97	2.79	0.44	0.06	0.35	0.40	-0.07	-0.03	-0.02	-0.89	-0.04	-0.18
-4.00 D	7.14	-1.40	3.14	0.39	0.09	0.27	0.22	0.00	-0.03	-0.01	-0.82	-0.03	-0.16
-5.00 D	7.67	-2.16	3.87	0.40	0.13	0.31	0.27	-0.01	0.01	-0.02	-0.78	0.03	-0.17
-6.00 D	8.19	-2.52	4.88	0.67	0.06	0.95	-0.26	0.00	-0.11	0.43	-1.12	0.09	0.04
-7.00 D	8.52	-3.49	6.41	0.77	0.21	1.77	0.00	0.07	0.13	0.21	-1.05	0.22	-0.07
-8.00 D	8.55	-4.89	8.08	1.09	0.04	2.90	-0.60	0.22	0.25	0.07	-0.87	-0.35	0.07

## Discussion

As usually observed in our past usage of the WASCA system, the measured area of WA for a group of myopic patients undergone excimer laser corrections in the current study were significantly smaller than their real pupil sizes as measured with the Pentacam system (**Table 2** and **Fig. 2**). The reduction of the measured area of WA, as compared to the real pupil size, was not random for individuals, but systematically related to the parameters of laser ablation (**Table 3**). More reduction of the measured area of WA was observed for the eye received laser ablation for greater myopia and with smaller OZ (**Table 3**). The mean measured area of WA was smaller than 6.0 mm for high myopic group (**Fig. 2**). An interesting finding was that the reduction of measured area of WA was correlated to postoperative corneal E value. The results therefore show that there is a systematic reduction of measured area of WA, as compared to the real pupil size, for patients received refractive surgery, especially for high myopes, by using the WASCA system to measure WA. The reduction of measurement area indicates that the real pupil area which is beyond the measured area was missed from the measurement, and thus the measured WA was underestimated. This point should be aware when we try to use wavefront aberrations to explain visual symptom experienced under scotopic visual condition for patients received refractive surgery.

The finding of systematic reduction of measured area of WA with Rx and ablation OZ could help us to understand the reason responsible for the problem of postoperative measurement area with Hartmann-Shack system. When a myopic refractive surgery is planned, the corneal tissues to be ablated are mathematically determined according to several variables including the refractive power to be corrected[20], optical zone[20], transition zone design[21], radius of the corneal curvature, and et al. But, the ablation pattern is calculated to correct refractive error of the pupil center. It doesn’t guarantee the refractive power to be corrected with the same amount for the whole pupil area. Theoretically, the corrected refractive power is supposed to be consistent only across the optical zone. For the transition zone which is between the optical zone and the peripheral unablated area, the corrected refractive power smoothly decreases towards the peripheral area. So, there is a great and fast change in refractive power across the transition zone. In practice, the optical zone is usually set at 6.0 mm or so, which could be smaller than the real pupil size especially for the scotopic visual condition. This will require the wavefront sensor to have very large measurement range in order to completely resolve the local wavefront aberrations for the whole pupil area when pupil size is large. But, the measurement range of the WASCA is limited due to the sharpness of the small image spots and filtering of optical noise in optical channel, so that the peripheral pupil area could not be successfully sampled. This might explain why the measured area is smaller than the real pupil size because the local refractive powers at pupil area outside the measured area were out of the measurement range of the WASCA.

By adjusting the spherical error compensation of the WASCA, the postoperatively measured area of WA was increased (**Fig. 3**). This might shift the limit of the measurement range up so that it covered the local refractive power on the pupil periphery. But the range shift was compensated by a loss of measurement accuracy of wavefront aberrations in the pupil center area (**Table 5**).

Overall, the problem of post-operative measurement area of WA in myopic laser surgery was investigated in this study, and the relationship between the reduction of the measured area of WA from the real pupil size and the ablation parameters suggest that the measurement range might be the cause for the limit in the WASCA system to measure the eyes with large variation in wavefront error over a large pupil area. In order to increase measurement range of the Hartmann-Shack wavefront sensor, further improvement in its design and manufacture will be desired.
